# Efficacy of Sonic-Powered and Manual Toothbrushes on Plaque-Induced Gingivitis in Pregnant Women: A Randomized Controlled Trial

**DOI:** 10.3390/mps6050099

**Published:** 2023-10-12

**Authors:** Céline Clément, Denis Bourgeois, Flavia Vitiello, Herve Perrier, Ariane Tabary, Florence Carrouel

**Affiliations:** 1 Laboratory “Interpsy”, UR4432, University of Lorraine, 54015 Nancy, France; celine.clement@univ-lorraine.fr; 2 Laboratory “Health, Systemic, Process” (P2S), UR4129, University Claude Bernard Lyon 1, University of Lyon, 69008 Lyon, France; denis.bourgeois@univ-lyon1.fr (D.B.); f.vitiello@pm.univpm.it (F.V.); 3 Department of Clinical Sciences and Stomatology (DISCO), Polytechnic University of Marche, 60126 Ancona, Italy; 4 Clinical Research Unit, Protestant Infirmary, 69004 Lyon, France; herve.perrier@infirmerie-protestante.com; 5 Majorelle Polyclinic, Elsan Group, 54000 Nancy, France

**Keywords:** pregnancy, prophylaxis, oral hygiene, periodontitis, gingival inflammation, microbiota, biofilm, toothbrush

## Abstract

Hormonal changes and physiological alterations in pregnancy increase the susceptibility of the woman to oral diseases such as plaque-induced gingivitis. In individual oral prophylaxis, effective tooth brushing can reduce gingival inflammation. Therefore, it is necessary to update the scientific evidence to identify which type of toothbrush, manual or sonic-powered, is most effective in reducing the incidence of gingivitis in pregnant women. The aim of this clinical trial is to compare the biofilm control effectiveness of two manual and two sonic toothbrushes in pregnant women. This study is designed as a four-arm, parallel, randomized controlled trial with an allocation ratio of 1:1:1:1. The pregnant woman will be included at 15–18 weeks of amenorrhea and followed for 3 months. The primary outcome will be the change in the incidence of gingival bleeding from a baseline and various follow-up periods of the study. Secondary outcomes measures will be to compare clinical effects of the toothbrushes tested on (i) gingival inflammation, (ii) dental plaque, (iii) gingival attachment and (iv) periodontal pocket; and to assess toothbrush acceptability. Thus, identifying the best device for effective tooth brushing in pregnancy could be helpful in reducing and improving the incidence of gingival inflammation.

## 1. Introduction

The World Health Organization visualizes a society where all pregnant women receive quality care during pregnancy, childbirth and postpartum periods [1]. Specifically, antenatal care constitutes a foundation for significant healthcare activities, such as health promotion and diagnosis, as well as disease prevention.

Plaque-induced gingivitis in pregnant women is a common inflammatory periodontal disease that appears from the second or third month of pregnancy with an overall prevalence of 30 to 100% [2,3,4,5,6]. Gingivitis during pregnancy can manifest as swelling, redness, increased volume and gingival sensitivity, but more especially through bleeding, indicating damaged vascularization [7]. Periodontal tissue inflammation as a result of oral biofilm dysbiosis significantly increases during a normal pregnancy, and is exacerbated by elevated levels of characteristic sex steroid hormones [8]. Indeed, inflamed periodontal tissues generate substantial quantities of proinflammatory cytokines, primarily interleukin 1-beta (IL-1β), IL-6, prostaglandin E2 and tumor necrosis factor-alpha (TNF-α), which could potentially exert systemic effects on the woman [9]. Periodontal pathogens, such as *Porphyromonas gingivalis*, can, especially in case of gingival bleeding, penetrate the bloodstream and potentially the placenta, with negative consequences for the pregnancy [10]. The research has affirmed a connection between periodontitis and certain pregnancy complications, including premature birth, low birth weight, intrauterine growth retardation and preeclampsia [11]. 

There is a consensus that adequate control of bacterial plaque, through professional and individual oral prophylaxis, is essential to maintain a symbiotic periodontal microbiota. Therefore, individual oral prophylaxis through standard oral hygiene methods, mainly based on the use of a mechanical toothbrush for the accessible surfaces, has a fundamental role in the prevention of plaque-induced gingivitis [12,13]. And if adequate toothbrushing associated with an effective, acceptable and tissue non-traumatizing toothbrush is the key to disorganizing dental and periodontal biofilms, certain coadjuvant products, such as gels containing natural antioxidant ingredients or others, can contribute to prevent or reduce gingivitis and/or aphthous lesions [14].

Mechanical plaque control involves many processes involving devices such as manual toothbrushes and so-called “intelligent” toothbrushes, which include powered toothbrushes, sonic and ultrasonic toothbrushes, ionic technology toothbrushes, disposable toothbrushes and laser toothbrushes. Continuous progress has been made with the integration of technology to improve the quality of the tools. However, numerous clinical studies have compared plaque removal and other oral health indices with powered and manual toothbrushes, but the results are controversial [15,16,17]. 

New generations of manual toothbrushes mainly based on a high increase in the number of bristles per tooth head would be likely to have optimum effectiveness in disrupting biofilms on accessible surfaces. This increase in the number of bristles is accompanied by a reduction in their diameter, which facilitates access to the gingival sulcus and the flexibility to prevent adverse effects in terms of dental and gingival tissue loss.

Powered toothbrushes, available at a wide range of prices, have been developed to take over the complex movements required of the manual toothbrush and make plaque removal more effective. A variety of powered toothbrushes with different toothbrush head shapes and different types of movement are marketed. Modes of action include counter-oscillation, rotation-oscillation, circular and sonic movements. The most convincing data seem to concern rotation-oscillation brushes, suggesting a more significant reduction in plaque and gingivitis than manual brushes [18,19]. However, sonic toothbrushes seem promising, as their use would be associated with a significantly greater reduction in plaque accumulation and gingival inflammation than toothbrushes with rotating-oscillating heads [20]. In addition, they are claimed to disrupt plaque microorganisms not only at the point where the bristles come into contact with the tooth surface, but also through non-contact energy transfer. The sonic models, called vibratory, vibrate between 15,000 and 30,000 times per minute, and are specifically designed to break up dental plaque through the action of the brush bristles, which vaporize saliva in the form of bubbles. Within sonic technology, different toothbrush head configuration options exist. The analogy with manual toothbrushes is real in terms of assumptions about the effectiveness, trauma and individual acceptability of these different models. The relevance of brush head models is currently debated. 

Furthermore, powered devices address the problem of the wide diversity of recommendations on tooth brushing methods. Similarly, the recent introduction on the market of interactive powered toothbrushes, thanks to the functionalities of applications associated with mobile phones, would provide better results in terms of plaque control and efficient use of brushing time, with better collaboration in patient self-treatment [21]. Interactivity is based on intelligent sensor technology with integrated pressure sensors that provide a warning in the event of brushing excess. Likewise, the brushing process is connected to the company’s application, using software compatible with Android and Bluetooth^®^ 4.0, and via the Bluetooth^®^ wireless application, a personalized progress report is sent back to the user. However, the overall effect size is limited, and the clinical relevance is not yet clear. Studies on the clinical efficacy of sonic brushes have so far been inconclusive.

To deliver on the “WHO Global Strategy for Women’s, Children’s and Adolescents’ Health”, innovative [22], evidence-based strategies for antenatal care are needed. In relation to the prevention and management of gingivitis, questions need to be asked about evidence-based practices during antenatal care that improve outcomes and lead to a positive pregnancy experience. Because of the potential difficulties of disorganizing dental biofilm in pregnant women such as nausea, acid reflux and vomiting, sensitive or bleeding gums, or fatigue [23,24], which can make it more difficult to maintain a regular oral care routine, powered toothbrushes have been advanced as an alternative to manual toothbrushes. In the literature, there are many studies on this topic [25,26,27], but there are currently no significant clinical trials investigating this long-term clinical comparison in pregnant woman. Therefore, the question of whether manual or powered toothbrushes are beneficial for pregnant women remains unresolved. In light of this, there is a need to update new scientific evidence to investigate the effectiveness of different toothbrushing models. Robust clinical research is required to assess their efficacy and acceptability in order to guide professional recommendations. Hence, our research will provide a significant advance in the understanding of the potential of the latest generation of powered toothbrushes to reduce gingival inflammation, and potentially reduce the risk of gingival lesions.

The aim of this randomized controlled clinical trial is, in pregnant women, to compare the efficacy on gingival inflammation of a new generation of connected sonic toothbrush with micro-vibrations, compared with a standard sonic toothbrush and two manual toothbrushes.

## 2. Experimental Design

### 2.1. Trial Design

**Figure 1:**
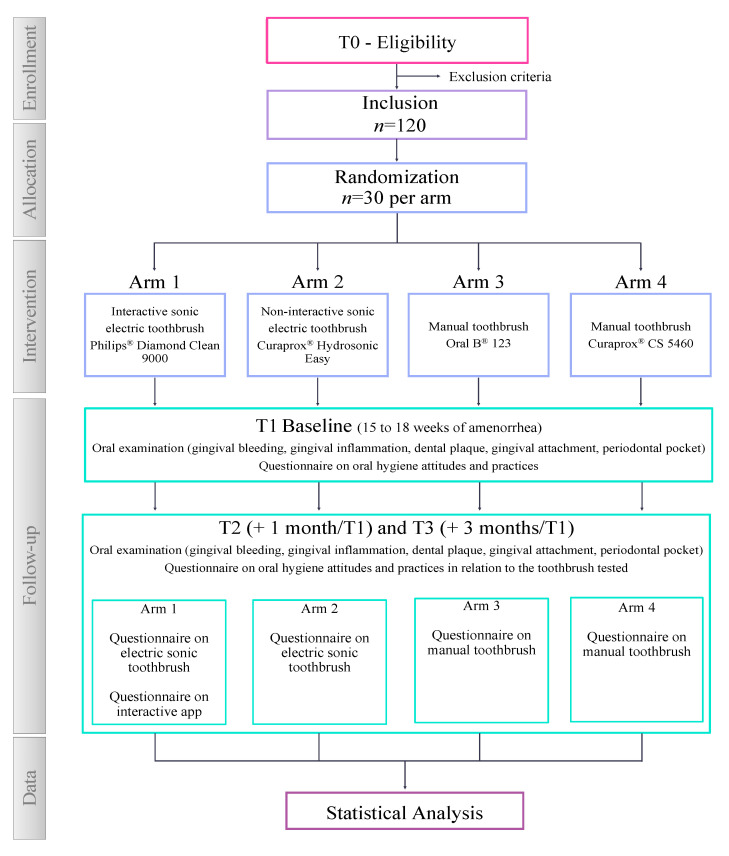
Flow chart diagram of this study. Philips^®^ (Suresnes, France), Curaden^®^ (Kriens, Switzerland), Procter & Gamble^®^ (Asnières-sur-Seine, France).

This clinical study named PRE-IOP (Pregnancy—Individual Oral Prophylaxis) is designed as a four-arm, parallel, randomized controlled trial (ratio 1:1:1:1) (Figure 1). The protocol is presented in accordance with Standard Protocol Items: Recommendations for Interventional Trials (SPIRIT) guidelines [28]. 

### 2.2. Study Setting

The PRE-IOP study will be conducted within the obstetrical department of a private hospital with a level 2B maternity unit, which is the highest level for a private hospital (Majorelle Polyclinic, Elsan group, Nancy, France).

### 2.3. Study Population

#### 2.3.1. Eligibility Criteria

For this clinical study, 120 pregnant women who meet the inclusion criteria will be recruited during their consultation visit with a medical specialist in obstetrics gynecology. During an initial oral examination, a dentist will verify the individual eligibility criteria: (i) female, (ii) 18–40 years old, (iii) pregnant 15–18 weeks amenorrhea, (iv) plaque-induced gingivitis with an intact periodontium or a reduced periodontium, (v) acceptance of study terms and conditions and (vi) signature of informed consent form.

#### 2.3.2. Exclusion Criteria

Exclusion criteria will be (i) women under guardianship; (ii) stage I, II, III periodontitis according to the classification system 2017 [29]; (iii) history or treatment of periodontal disease; (iv) current dental treatment or orthodontic treatment; (v) less than 20 natural teeth, excluding third molars; (vi) taking medication affecting the gum and/or oral mucosa such as anticoagulant therapy, antiplatelet therapy and calcium channel blockers; (vii) use of interdental brushes and/or dental floss and/or mouthwashes; (viii) removable prosthesis; (ix) dental implants; (x) systemic disorder such as hematological disorders, diabetes and risk of infective endocarditis; and (xi) inability to answer questions or non-cooperation.

### 2.4. Materials Description

The materials used in this study are detailed in Table 1. According to manufacturers’ guidelines, sonic toothbrush heads should be replaced every 3 months due to bristle wear. Therefore, new sonic toothbrushes heads will be supplied to participants every 3 months. Similarly, manual toothbrushes will be replaced every 3 months.

### 2.5. Interventions

During the study, the pregnant women will have to brush their teeth twice a day (morning and evening) with the toothbrush corresponding to the arm of randomization assigned (Table 1). All participants will use the same toothpaste 0.243% SnF_2_ (Sensodyne rapid action toothpaste, GSK, London, UK) and they will not be allowed to use complementary techniques (dental floss, interdental brushes, mouthwashes, etc.) during the period of the study. Technical documentation on the use of the devices produced by the manufacturer will be made available to participants. No individual instructions on brushing hygiene techniques will be provided. This trial does not interfere with the organization of care provided during obstetrical visits.

### 2.6. Outcomes

#### 2.6.1. Primary Outcome Measures

Primary outcome will be the change in the incidence of gingival bleeding in pregnant woman (time frame: 3 months).

#### 2.6.2. Secondary Outcome Measures

Secondary outcome measures will compare the effectiveness of the toothbrushes tested on the clinical changes in (i) gingival inflammation, (ii) dental plaque, (iii) gingival attachment and (iv) periodontal pocket (time frame: all outcomes will be measured at baseline (T1), 1 month/T1 (T2) and 3 months/T1 (T3))

Secondary outcomes measures will also be to evaluate (i) oral hygiene attitudes and practices (time frame: baseline (T1)), (ii) attitudes and practices in relation to the toothbrush tested (time frame: 1 month/T1 (T2) and 3 months/T1 (T3)) and (iii) assessment of the acceptability of the toothbrush tested (time frame: 1 month/T1 (T2) and 3 months/T1 (T3)).

### 2.7. Timeline of Participant

The timing of visits will coincide with the usual follow-up visits required for pregnant women. The trial participation schedule is detailed in Table 2.

#### 2.7.1. Pre-Screening and Eligibility Assessment (T0)

Participants will be selected during their gynecologist visit for pregnancy. The obstetrician will present the study to all pregnant women eligible. If pregnant women accept to participate and consent to be screened, an inclusion visit will be scheduled by a dentist. 

#### 2.7.2. Inclusion, Oral Examination and Questionnaire (T1)

Participants will sign the informed consent form. They will have to complete a questionnaire regarding their oral hygiene attitudes and practices (Supplementary File S1) and an oral examination in accordance with clinical practices will be carried out by a dentist who will report the following clinical parameters:

(i) Bleeding on probing (BOP): Dichotomous gingival index reporting the presence/absence of bleeding on probing after 30 s (0 = absence of bleeding after 30 s, and 1 = presence bleeding after 30 s). Four sites are recorded per tooth (mesio-buccal, disto-buccal, mesio-palatine and disto-palatine) [30,31].
(ii) Gingivitis score (Gingival Index, GI): Measured through visual observation from 0 to 3 (0 = no inflammation; 1 = slight change, slight inflammation in color and little change in texture; 2 = moderate, moderate inflammation, redness, edema and hypertrophy, tendency to bleed on probing; 3 = severe, marked redness, inflammation and hypertrophy; tendency to spontaneous bleeding). Gingivitis score = sum of GI scores divided by number of total sites [32].
(iii) Dental plaque score (Rustogi Modified Navy Plaque Index, RMNPI): According to the Navy plaque index modified by Rustogi et al., the presence of plaque deposits is sought on the vestibular and lingual surfaces of the teeth. Each tooth face is divided into 9 areas to which a dichotomous score is assigned (0 = absence of plaque; 1 = presence of plaque). Thus, for each tooth, 18 measurements are carried out. This index makes it possible to detect minimal differences at the partial level of the marginal/interproximal zones, or at the total level of the oral cavity [33].
(iv) Probing pocket depth (PPD) score: Measure indicating the distance separating the top of the marginal gingiva from the bottom of the periodontal pocket. The measurement is expressed in millimeters. Four sites are recorded per tooth (mesio-buccal, disto-buccal, mesio-palatine and disto-palatine) [34].
(v) Clinical Attachment Loss (CAL) score: Addition of PPD and recession height, which is the distance separating the enamel–cementum junction from the bottom of the pocket. Four sites are recorded per tooth (mesio-buccal, disto-buccal, mesio-palatine and disto-palatine) [31].

PPD and CAL will be assessed following the criteria defined by the consensus report of the 2017 World Workshop on the Classification of Periodontal and Peri-Implant Diseases and Conditions [29], using U.S. Williams PDT sensor probe at a pressure of 20 g (Zila-Pro-Dentec Inc., Batesville, AR, USA). Based on severity-defined stages of interdental CAL and tooth loss, and complexity, extent and distribution, they included a full oral examination of six sites on each permanent tooth. All measures will be performed on all teeth except for third molars. No radiographs will be taken, for ethical reasons.

Four calibrated, properly trained dentists, blinded to group category at the time of the assessment, will perform the periodontal evaluations of all participants. Intraclass correlation coefficients for PPD and CAL will be determined at the level of the site. The intra- and inter-examiner coefficients for CAL will be, respectively, from 0.80 and 0.85, and from 0.75 and 0.85 for probing depth.

#### 2.7.3. Follow-Up Visits (T2 and T3)

Two visits will be made after the inclusion of pregnant woman at T1. Visit T2 will occur one month after T1 and visit T3 will occur three months after T1 (Table 2). At each visit, an oral clinical examination and a questionnaire regarding oral hygiene attitudes and practices in relation to the toothbrush used, specific for each arm of the study (Supplementary Files S2–S5), will be performed. 

### 2.8. Ethics Statement

The PRE-IOP protocol and design were approved by ethical and regulatory authorities, and will be performed in conformity with the Declaration of Helsinki. The Committee for the Protection of Persons Ile de France II (Paris, France) approved the protocol on 3 July 2023. The National Agency for Medical and Health Product Safety registered it on 18 April 2023 (ID-RCB ref: 2022-A02433-40). This study will be conducted in accordance with the methodology of reference MR-001 from National Commission for Information Technology and Liberties (2116544 v 0). This trial was registered with ClinicalTrials.gov, accessed on 7 June 2023 (identification number NCT05945225).

Before taking part in the study, all participants should provide informed consent. The following elements will be included in the consent form: the name and affiliation of principal investigator, a clear description of the purpose of the study, the procedure and the duration of the study, the right to stop at any time, the approval of the ethics committee and a confidentiality guarantee. 

### 2.9. Sample Size

The reduction in gingival bleeding was considered the primary outcome variable and an estimate of a mean difference in reduction was used to calculate the sample size, when the two-sided differences in means between groups (treatment effect) were 5% (estimation extracted from ongoing project in pregnant women in Senegal; unpublished data), with a standard deviation of 10%. Using this estimate with an alpha risk of 5% and statistical power of 90% resulted in the required number of subjects per group of 25. Assuming a potential dropout rate of 20%, 30 participants per group were determined as a target for patient inclusion.

## 3. Procedure

### 3.1. Recruitment

The PRE-IOP study will recruit 120 pregnant female volunteers. The obstetrician will propose the study to all women between 15 and 18 weeks of amenorrhea and between 18 and 40 years of age. If the pregnant woman accepts to be included in the study, then the obstetrician will give her a trial information sheet and a consent form, and an inclusion visit will be planned with a dentist. The total number of pregnant women screened and recruited will be referenced. Participants will not receive any financial compensation.

### 3.2. Allocation of Interventions

#### 3.2.1. Allocation

In the PRE-IOP trial, participants will be randomly assigned at the start of the trial to one of four arms: Arm 1—Interactive sonic toothbrush, DiamondClean 9000 Sonicare (Philips^®^, Suresnes, France); Arm 2—Non-interactive sonic toothbrush, HydroSonic Easy (Curaden^®^, Kriens, Switzerland); Arm 3—Manual toothbrush, Oral-B 123 (Procter & Gamble^®^, Asnières-sur-Seine, France); and Arm 4—Manual toothbrush Curaprox CS 5460 (Curaden^®^, Kriens, Switzerland)) (Table 1). Random arm allocation will be computer-generated by the study manager using e-CRF Voozalyon 1.3 (Voozanoo, Caluire, France). Participants will remain in the same arm during the study period. Eligible pregnant women will be included in the study and numbered according to the inclusion order up to the number of subjects (*n* = 120, 30 pregnant women per group).

#### 3.2.2. Blinding

There will be a clear distinction between the assignment generator and the individuals responsible for carrying out the assignments. Intervention implementation will be conducted by staff who are not implicated in the process of data collection. To ensure impartiality, during the assessment and analysis phase, statisticians, clinical research associates and clinicians will not know the group to which the participant belongs. The identification codes will be securely held by the study monitor and will remain sealed until the conclusion of the study to maintain confidentiality and prevent bias.

### 3.3. Data Collection, Management and Analysis

#### 3.3.1. Data Collection Methods

An eCRF, specific to the PRE-IOP study, will be created by a data manager from ArcaLyon (Lyon, France). The software used will be the ECRF ‘EVOOZ’, version 4.90, hosted on an HDS server located in France. This software conforms to the recommendations of the FDA on computerized systems for the management of clinical trials (Guidance for Computerized Systems Used in Clinical Trials) and FDA recommendations on electronic signature (21CFR part 11). Only data required for the protocol and scientific publication will be recorded in the eCRF. Other patient information required for follow-up outside this study will be recorded in the medical dossier. This e-CRF will assign to each pregnant woman a unique identification code that will permit the labeling of all documents, forms and data files. Thus, data recorded by investigators for each participant will be anonymized according to the Data Protection Act. As data are collected, it must be completed by authorized persons (investigators) with their own identifiers, according to the Data Protection Act. During the entry, the data are immediately checked thanks to consistency checks. The person in charge of filing must validate and justify any value change in the eCRF. Entries and modifications are subject to an audit trail.

The quality, precision and relevance of all input data will be the responsibility of the investigator. As such, each page of the patient’s eCRF should be dated and signed electronically by the investigator to signify their agreement and their responsibility regarding the data collected.

#### 3.3.2. Data Management

Experimental and personal data will be analyzed using a unique identification code, ensuring the exclusion of any personal characteristics. The subjects could be identified using a subject re-identification file, which will be securely maintained solely at the clinical study site. The signed informed consent documents will also be kept exclusively at the clinical study site for confidentiality and privacy purposes.

#### 3.3.3. Data Analysis Methods

Statistical analysis will include the production of descriptive data summaries, modeling of data with a mixed (linear) model and assessment of correlations between bleeding rates. SPSS 12.0 (SPSS Inc., Chicago, IL, USA) will be used to calculate descriptive statistics (means, standard deviations and percentages). For repeated measurements at different times during the study, the SUDAAN 7.0 test (Research Triangle Institute, Research Triangle Park, NC, USA) will be used to perform statistical tests (*p*-values). The mixed linear model to explain the evolution of the quantification of bleeding over time as a function of certain independent variables first requires working on the log transform of the quantifications of bleeding. Then, in the linear mixed model, the estimated coefficients are tested for each independent variable, with the null hypothesis being that the estimated coefficient is equal to zero, at the threshold of 0.05. The fixed effects are all the independent variables that we want to test (age, CSP, experimental group, etc.), with the random effect being the subject factor, on which the four clinical bleeding probing analyses are repeated.

## 4. Expected Results

The aim of the PRE-IOP randomized controlled trial is to compare the biofilm control effectiveness in pregnant women with four different toothbrushes: two manual toothbrushes and two sonic toothbrushes. The primary endpoint is the evolution in gingival bleeding over the different study periods. In addition to these clinical data, questionnaires on the acceptability of the toothbrushes tested will be analyzed to determine which of the toothbrushes tested is most acceptable to pregnant women. Clinical relevance will allow us to ensure that the outcome of the trial corresponds to a sufficiently large effect on a clinically relevant endpoint. We expect to observe significant differences in the efficiency of the four toothbrushes in disorganizing oral biofilm. One or more of the toothbrushes could prove more effective than the others, which would suggest that the choice of toothbrush has an impact on the reduction in oral biofilm.

These results would support the hypothesis that selecting the appropriate toothbrush, with the adequate brushing technique, can contribute to improving patients’ oral health. Also, these outcomes will provide decision-making support for healthcare professionals involved or not in oral health, to recommend the best devices.

## Figures and Tables

**Table 1:** Description of the materials used in PRE-IOP randomized controlled trial and the randomization in four arms.

Arm	Arm 1	Arm 2	Arm 3	Arm 4
**Product name**	Diamond Clean 9000 Sonicare	Hydrosonic Easy	Oral-B^®^ 123	CS 5460
**Type of** **toothbrush**	Interactive sonic	Non-interactive sonic	Manual	Manual
**Bristles** **hardness**	Soft	Ultra-soft	Medium	Ultra-soft
**Filaments** Material, diameter	Nylon, <0.18 mm	Curen^®^, 0.1 mm	Not available	Curen^®^, 0.1 mm
**Manufacturer** Name (city, country)	Philips^®^ (Suresnes, France)	Curaden^®^ (Kriens, Switzerland)	Procter & Gamble^®^ (Asnières-sur-Seine, France)	Curaden^®^ (Kriens, Switzerland)

**Table 2:** Trial participation schedule.

Date of Pregnancy Procedures/Visits		Timeline		
	T0	T1	T2	T3
		15–18 Weeks ^a^	+1 Month/T1	+3 Months/T1
Pre-screening	X			
Eligibility screening	X			
Informed consent	X			
Questionnaires		X	X	X
*Oral hygiene attitudes and practices* **^b^**		X		
*Oral hygiene attitudes and practices in relation to toothbrushes tested* **^c^**			X	X
*Acceptability of the toothbrush* **^d^**			X	X
Oral examination		X	X	X
*Bleeding on probing*		X	X	X
*Gingival Index*		X	X	X
*Plaque Index*		X	X	X
*Probing Pocket Depth*		X	X	X
*Clinical Attachment Loss*		X	X	X

**^a^** Weeks of amenorrhea. **^b^** Supplementary File S1. **^c^** Supplementary File S2. **^d^** Questionnaire in accordance with the arm of the randomization. Arm 1: Supplementary File S3 and S4. Arm 2: Supplementary File S3. Arm 3 and 4: Supplementary File S5.

## Data Availability

No new data were created or analyzed in this study. Data sharing is not applicable to this article.

## Supplementary Materials

The following supporting information can be downloaded at https://www.mdpi.com/article/10.3390/mps6050099/s1. Supplementary files: PRE-IOP study questionnaires. Supplementary File S1. Questionnaire regarding oral hygiene attitudes and practices for all pregnant women at T1 (baseline: 15–18 weeks of amenorrhea). Supplementary File S2. Questionnaire regarding oral hygiene attitudes and practices in relation to the toothbrush tested for all pregnant women at T2 (1 month after T1) and T3 (3 months after T1). Supplementary File S3. Questionnaire for pregnant women using sonic toothbrush (arms 1 and 2) at T2 (1 month after T1) and T3 (3 months after T1). Supplementary File S4. Questionnaire only for pregnant women using interactive sonic toothbrush (arm 1) at T2 (1 month after T1) and T3 (3 months after T1). Supplementary File S5. Questionnaire for pregnant women using manual toothbrush (arms 3 and 4) at T2 (1 month after T1) and T3 (3 months after T1).
