# Supplementary material for: Efficacy of Sonic-Powered and Manual Toothbrushes on Plaque-Induced Gingivitis in Pregnant Women: A Randomized Controlled Trial
Source: Methods Protoc. 2023 Oct 12;6(5):99. doi: 10.3390/mps6050099 (PMC10608920; doi:10.3390/mps6050099)
Supplement: Supplementary file 1 [file mps-06-00099-s001.zip › mps-2601799-supplementary.pdf]

Supplementary Material.
PRE-IOP study questionnaires

Supplementary File S1. Questionnaire regarding oral hygiene attitudes and practices for all pregnant women at T1 (Baseline: 15-18 weeks of amenorrhea)

1 - How old are you? |__|__| years

2 - What is your level of study?
❑ < Baccalaureate ❑ > Baccalaureate

3 - What is your occupation? ______________________________

4 - How many children do you have? ______________________________

5 - How would you describe your general health today?
❑ Good ❑ Average ❑ Poor ❑ Very poor

6 - How would you describe your health dental today?
❑ Good ❑ Average ❑ Poor ❑ Very poor

7 - How would you describe your gum health today?
❑ Good ❑ Average ❑ Poor ❑ Very poor

8 - Did your gums bleed before your pregnancy?
❑ Yes ❑ No

9 - Are your gums currently bleeding?
❑ Yes ❑ No

10 - How many times do you brush your teeth per day (number of times/day)?
❑ 1 times/day ❑ 2 times/day ❑ 3 times/day ❑ More than 3 times/day

11 - How long do you currently brush (brushing time in minutes)?
❑ Less than 1 minute ❑ 1 to 2 minutes ❑ 3 to 5 minutes ❑ More than 5 minutes

12 - What type of toothbrush do you use?
❑ Manual toothbrush ❑ Electric/sonic toothbrush ❑ Both types
12.1 – Which brand?______________________________ ❑ Don't know

13 - What type of toothbrush head do you use?
❑ Hard ❑ Medium ❑ Soft ❑ Ultrasoft

14 - How often do you replace your toothbrush or its head (in months)?
❑ 1 to 2 months ❑ 3 to 5 months ❑ 6 or more ❑ Don't know ❑ Variable

15 - What are the criteria for buying your toothbrush?
❑ Dentist advice ❑ Pharmacist advice ❑ Large surface advisor ❑ Price
❑ Publicity ❑ Internet ❑ Design/ Aesthetics ❑ Efficiency
❑ Advice from family, friends ❑ No criteria ❑ Other

16 - Do you always use the same brand and type of toothbrush?

❑ Yes ❑ No

17 - Where do you buy your toothbrush?

❑ Large surface – Shops ❑ Internet ❑ Pharmacy ❑ Other, specify________

18 - When was your last visit at the dentist?

❑ During my pregnancy ❑ Less than 1 year ❑ 1 year to 2 years ❑ More than 2 years

19 - What was the reason for the last visit to the dentist? (if <2 years)

❑ Control ❑ Pain/Emergency ❑ Prevention ❑ Care

20 – Since the beginning of your pregnancy:

❑ You brush your teeth more often ❑ You brush less often the teeth

❑ You use interdental brushes ❑ You use mouthwashes

21- Have you carried out or made an appointment to carry out the dental consultation recommended by social security during pregnancy?

❑ Yes ❑ No

<u>Supplementary file S2.</u> Questionnaire regarding oral hygiene attitudes and practices in relation to the toothbrush tested for all pregnant women at T2 (1 month after T1) and T3 (3 months after T1)

1- How many times did you brush your teeth per day (number of times/day) with the new toothbrush offered?

❑ 1 time/day ❑ 2 times/day ❑ 3 times/day ❑ More than 3 times/day

2- Have you used this brush?

❑ Only once in a while ❑ Never

3- If occasionally or never, can you tell us why? ______________________________________

4- What is the current duration of your brushing (duration of brushing in minutes)?

❑ Less than 1 minute ❑ 1 to 2 minutes ❑ 3 to 5 minutes ❑ More than 5 minutes

5 – Have you consulted a dentist since our last interview?

❑ Yes ❑ No

6- If yes, what was the reason?

❑ Control ❑ Pain/Emergency ❑ Prevention ❑ Care

7- Have you carried out or made an appointment to carry out the dental consultation recommended by social security during pregnancy?

❑ Yes ❑ No

<u>Supplementary file S3.</u> Questionnaire for pregnant women using electric sonic toothbrush (arms 1 and 2) at T2 (1 month after T1) and T3 (3 months after T1)

Please indicate your level of agreement with each of the following statements:

	1 = Strongly disagree						7 = Strongly agree
**1- With this toothbrush, I have the feeling of cleaning my mouth easily**	❑	❑	❑	❑	❑	❑	❑
**2- With this toothbrush, I have the feeling of cleaning my mouth thoroughly/effectively**	❑	❑	❑	❑	❑	❑	❑
**3 – With this toothbrush have you the feeling of cleaning your mouth hurting yourself**	❑	❑	❑	❑	❑	❑	❑

	1 = Strongly dissatisfied						7 = Extremely satisfied
**4 – In terms of aesthetics what mark out of 7 would you give to this toothbrush?**	❑	❑	❑	❑	❑	❑	❑
**5- In terms of handling, what mark out of 7 would you give to this toothbrush?**	❑	❑	❑	❑	❑	❑	❑
**6- On a scale of 1 to 7, how satisfied are you with:**	❑	❑	❑	❑	❑	❑	❑
6.1- Comfort?	❑	❑	❑	❑	❑	❑	❑
6.2- The pleasure of use?	❑	❑	❑	❑	❑	❑	❑
6.3- Perceived effectiveness?	❑	❑	❑	❑	❑	❑	❑
6.4- Noises and vibrations?	❑	❑	❑	❑	❑	❑	❑
6.5- Functions and accessories?	❑	❑	❑	❑	❑	❑	❑
**7- What overall score out of 7 would you give to this toothbrush?**	❑	❑	❑	❑	❑	❑	❑

	1 = categorically no						7 = totally yes
**8- In the end, do you prefer this toothbrush to the one you used before?**	❑	❑	❑	❑	❑	❑	❑

9- What do you like most about this new toothbrush?
Free answer: ____

10- If you could improve anything, what would it be?
Free answer: ____

	1 = Extremely unlikely / difficult						7 = Extremely likely / easy
**11- On a scale of 1 to 7, how likely are you to buy this toothbrush again?**	❑	❑	❑	❑	❑	❑	❑
**12- On a scale of 1 to 7, did you find using this new toothbrush easy?**	❑	❑	❑	❑	❑	❑	❑
**13- On a scale of 1 to 7, how likely are you to recommend this toothbrush to someone you know?**	❑	❑	❑	❑	❑	❑	❑
**14- On a scale of 1 to 7, to what extent will you continue to use the toothbrush in the future?**	❑	❑	❑	❑	❑	❑	❑

15-Do you plan to use this new toothbrush alternately with the one used before the study?

❑ Yes ❑ No ❑ Don’t know

16- Do you have any comments or incidents to report about this toothbrush?

Free answer: ____

Supplementary file S4. Questionnaire only for pregnant women using interactive electric sonic toothbrush (arm 1) at T2 (1 month after T1) and T3 (3 months after T1)

Please indicate your level of agreement with each of the following statements:

1- Have you used the smartphone application?

❑ Yes* ❑ No** ❑ Don't know

****If not why? ____**

***If you have used the application, complete the following questionnaire**

	1 = Extremely unlikely / difficult						7 = Extremely likely / easy
**2- On a scale of 1 to 7, was the application easy to understand?**	❑	❑	❑	❑	❑	❑	❑

3- If it was difficult to understand, why?
Free answer:____

	1 = categorically no						7 = totally yes
**4- On a scale of 1 to 7, do you see yourself using the app in the future?**	❑	❑	❑	❑	❑	❑	❑
**5- On a scale of 1 to 7, would you recommend the app to friends or relatives?**	❑	❑	❑	❑	❑	❑	❑
**6- On a scale of 1 to 7, did you feel that the application gave you a better cleaning result?**	❑	❑	❑	❑	❑	❑	❑
**7- On a scale of 1 to 7, has using the app caused any changes in the way you clean your teeth?**	❑	❑	❑	❑	❑	❑	❑

8- What changes have you made?
Free answer:____

	1 = categorically no						7 = totally yes
**9- On a scale of 1 to 7, did you have difficulty using the application?**	❑	❑	❑	❑	❑	❑	❑

10- If yes, what were these problems?
Free answer:____

11- Do you have any comments on this Philips Application?

Free answer:____

Supplementary file S5. Questionnaire for pregnant women using manual toothbrush (arms 3 and 4) at T2 (1 month after T1) and T3 (3 months after T1)

Please indicate your level of agreement with each of the following statements:

	1 = Strongly disagree / dissatisfied						7 = Strongly agree / satisfied
**1- With this toothbrush, I have the feeling of cleaning my mouth easily**	❑	❑	❑	❑	❑	❑	❑
**2- With this toothbrush, I have the feeling of cleaning my mouth thoroughly/effectively**	❑	❑	❑	❑	❑	❑	❑
**3- With this toothbrush have you the feeling of cleaning your mouth hurting yourself**	❑	❑	❑	❑	❑	❑	❑
**4- In terms of aesthetics what mark out of 7 would you give to this toothbrush?**	❑	❑	❑	❑	❑	❑	❑
**5- In terms of handling, what mark out of 7 would you give to this toothbrush?**	❑	❑	❑	❑	❑	❑	❑
**6- In terms of comfort, what mark out of 7 would you give to this toothbrush?**	❑	❑	❑	❑	❑	❑	❑
**7- In terms of pleasure of use, what mark out of 7 would you give to this toothbrush**	❑	❑	❑	❑	❑	❑	❑
**8- What overall score out of 7 would you give to this toothbrush?**	❑	❑	❑	❑	❑	❑	❑
**9- In the end, do you prefer this toothbrush to the one you used before?**	❑	❑	❑	❑	❑	❑	❑

10- What do you like most about this new toothbrush?
Free answer: ______________________________

11- If you could improve anything, what would it be?
Free answer: ______________________________

	1 = Extremely unlikely / difficult						7 = Extremely likely / easy
**12- On a scale of 1 to 7, how likely are you to buy this toothbrush again?**	❑	❑	❑	❑	❑	❑	❑
**13- On a scale of 1 to 7, did you find using this new toothbrush easy?**	❑	❑	❑	❑	❑	❑	❑
**14- On a scale of 1 to 7, how likely are you to recommend this toothbrush to someone you know?**	❑	❑	❑	❑	❑	❑	❑
**15- On a scale of 1 to 7, to what extent will you continue to use the toothbrush in the future?**	❑	❑	❑	❑	❑	❑	❑

16- Do you plan to use this new toothbrush alternately with the one used before the study?

❑ Yes ❑ No ❑ Don't know

17- Do you have any comments or incidents to report about this toothbrush?

Free answer: ____
